# Design and development of ultra-broadband THz metamaterial MIMO antenna with efficient diversity parameters optimized with machine learning for TWPAN applications

**DOI:** 10.1038/s41598-026-40351-7

**Published:** 2026-02-24

**Authors:** Meshari Alsharari, Yogesh Sharma, Khaled Aliqab, Ammar Armghan, S. K. Patel, Aymen Flah

**Affiliations:** 1 Department of Electrical Engineering, College of Engineering, Jouf University, Sakaka, 72388 Saudi Arabia; 2 Department of Physics & Environmental Sciences, Sharda School of Engineering & Science, Sharda University, Greater Noida, Uttar Pradesh 201310 India; 3 Department of Computer Engineering- AI & Big Data, Marwadi University, Rajkot, Gujarat 360003 India; 4 National school of engineering of gabes, University of gabes, gabes, 6072 Tunisia; 5 ENET Centre, CEET, VSB-Technical University of Ostrava, Ostrava, 708 00 Czech Republic; 6 Applied Science Research Center, Applied Science Private university, Amman, 11931 Amman Jordan

**Keywords:** MIMO, TWPAN, Antenna, Machine learning: THz, Diversity parameters: high gain, Ultrabroad bandwidth, Engineering, Mathematics and computing

## Abstract

The high-speed communication development is revolutionizing the way with interact with technology by enabling ultra-fast and intelligent connectivity. There is a need for antenna design that operates with ultrabroadband in the THz regime to be applicable for Terahertz Wireless Persona Area Network (TWPAN) applications. We have proposed an ultra-fast, broadband, and high-gain MIMO antenna design which not only smart but also small in size and low cost to be considered for high-speed communication applications. The designed antenna shows a high gain of 15.7 dBi. The ultrabroadband response gives a bandwidth of 20 THz. The MIMO diversity parameters show the ECC value near 0 and DG of 10 dB. The CCL values are also 0.0083 bits/Hz. Their values show that there is minimal correlation, which means better MIMO performance. The performance is also optimized using parametric optimization and machine learning optimization. The machine learning algorithm gives the highest R^2^ value of 0.99, which gives a minimum prediction error and higher antenna performance. The THz metamaterial design with optimum diversity parameters makes it a good candidate for TWPAN applications.

## Introduction

MIMO antennas are the right choice for modern communication systems because of their capabilities, like spatial multiplexing, diversity gain, beamforming, and interference mitigation. The MIMO antenna is good for data transmission capacity, which is capable of handling numerous data streams over a frequency band. The MIMO antenna parameters and their real-world applications, which include modern wireless communication, are given in^1^. The review shows the fundamentals of MIMO systems, defining performance metrics and highlights the robust MIMO design^2,3^. The dual-band, Ultrawideband designs are investigated and compared in detail in that review. The spatial multiplexing improves spectral efficiency, which makes it suitable for high and fast data rates^4^. These fast data rates are crucial for today’s fast data speed requirements of many applications. The quadport MIMO design is investigated by researchers for the GHz frequency band in^5^. The design is integrated with a Jerusalem-cross absorber to suppress mutual coupling between antenna elements. This absorber significantly enhances isolation, and it also simplifies and compacts the design by improving impedance match and reducing coupling. The design is applicable to modern wireless communication systems, including 5G and beyond^6–9^. MIMO antenna also improves the signal reliability and coverage with diversity gain, which decreases fading effects. These MIMO performance makes it ideal for high-capacity base stations, mobile devices, mobile vehicles and IoT devices^10^. Improvement in the MIMO designs can make it suitable for the high-speed communication requirement. This improvement can be done by applying different materials as well as by applying optimization techniques.

The high-speed communication led to the design of an antenna with higher gain, bandwidth, and good diversity parameters. MIMO antennas can be designed with all these parameters, but there is a need for metamaterial components to be added to improve the parameters of the antenna design^11^. Graphene is also one of the materials that can be used for tuning the spectrum. The performance of THz MIMO antennas is enhanced using graphene material in^12^, behaviour of the antenna design is predicted, and this technique optimizes the antenna to be applicable to high-speed communication. In recent research, Kavitha et al. designed a graphene-based antenna for IoT applications operating at the THz spectrum^13^. They emphasize the advantages of using graphene in antenna design, which makes it suitable for optimum performance of the antenna. The design is also showing multiple bands, enabling enhanced spectral response and seamless integration to IoT devices. Metamaterial research for MIMO antenna show high gain response as given in^14^. The authors show a graphene patch placed over a SiO_2_ wafer based on a gold surface. The design is giving a good gain of 4.87 dBi and 0.15 THz bandwidth, which can be applied for high-speed networks. One drawback of the design is its cost-effectiveness.

Metamaterial MIMO antennas are important because of the metamaterial properties, which give an extra edge to the normal MIMO antennas^15^. Koch-fractal MIMO antenna designed with metamaterial design to be applicable in vehicular communications^16^. The metamaterial design is investigated for single, dual, and quad element designs to observe its effect. The metamaterials are applied with two distinctive metamaterial unit cells^17^. The improvement in the parameters is achieved because of the metamaterial properties. The higher isolation of -45dB is achieved using a metamaterial design^18^. Francis et al. introduced a metamaterial superstrate design that is applicable for sub-6 GHz applications. Metamaterial unit cells are included in the design, giving negative permeability results. These inclusions improve the gain by 5 dBi. The design is also showing strong bandwidth coverage of 2.35 GHz using this metamaterial design. Design is also validated by measured results showing good gain, bandwidth and diversity parameters^19^. The metamaterial compact MIMO design with sub-6 GHz, Ku-band response is presented in^11^. The design is showing the good results with a high gain of 6.97 dB, 0.007 ECC, and 9.96 dB DG. The design uses using defected ground structure to improve the surface current and improve isolation. The DGS and microstrip decouplers improved the isolation and gave robust MIMO antenna performance. MIMO antenna design is presented with mu-near-zero metamaterial. The design is achieving a good bandwidth of 3.35 GHz with a robust gain of 7.1dBi. The radiation efficiency of 68% with an isolation of 35dB is achieved for this research. The design is compact and wideband and applicable for sub-6 GHz 5G applications. This study shows that metamaterials can improve the performance of 5G communication MIMO antenna designs and can be used for 5G systems^20^. The multiple-element MIMO antenna design for 5G communication is presented in^21^. This design is implemented using a metamaterial architecture comprising complementary metamaterial screens, which gives near-zero refractive index results in the investigated range. This design gives results of high isolation and bandwidth control. The design is giving good isolation of 35 dB and a gain of 19.5 dBi. The performance enhancement is achieved using metamaterial integration, which gives 1 GHz bandwidth, robust gain and isolation ideal for 5G applications.

Machine learning (ML) is another important way to improve the antenna performance by optimizing its parameters. Antenna parameters like gain, bandwidth, etc. can be improved using machine learning optimization. A novel approach is introduced for the design and optimization of pattern reconfigurable antennas using ML algorithms. The work addresses the growing demand for adaptable and intelligent antenna systems, particularly for applications in 5G and next-generation wireless networks, where beam steering and radiation pattern control are critical^22^. M. A. Haque et al. presented a comprehensive design of a Quasi-Yagi antenna specifically tailored for LTE (Long-Term Evolution) applications, integrating ML methods to enhance design accuracy and performance prediction. The research focuses on leveraging data-driven models to estimate critical antenna parameters—namely gain and directivity—which are vital for assessing antenna effectiveness in wireless communication systems^23^. M. A. Haque et al., explored the design of a compact dual-band antenna and integrate ML techniques to accurately predict its resonance frequencies. This research aims to streamline the antenna design process by reducing dependency on time-consuming full-wave electromagnetic simulations, particularly for multiband applications in modern wireless communication systems^24^. Nan et al., presented an innovative methodology for the design and optimization of Ultra-Wideband (UWB) antennas using advanced machine learning models—specifically an improved Deep Belief Network (DBN) and an Extreme Learning Machine (ELM). The work addresses the complexity and high computational cost of traditional full-wave simulations in UWB antenna design by developing efficient surrogate models to predict key performance parameters^25^. The THz wireless application-based MIMO antenna has been presented by incorporation of graphene material with 13.67 dB of realized gain in operating range of 0.74 THz to 10 THz^26^. The sunshine-shaped MIMO antenna for 6G application in THz spectrum range of 3.6, 4.5, 5.2, and 6.2 THz with a high gain value of 8.5 to 9.2 dB^27^. The four port MIMO antenna with the size of 120 μm × 120 μm, which operates at multiband of 1.750 THz, 2.590 THz, 4.480 THz, and 7.410 THz along with machine learning analyzation^28^. A patch antenna design of MIMO antenna for THz application in the band of 3.572 to 3.6818 THz with 50 dB isolation enhancement^29^.

A comparative analysis has been done of THz antenna with the use of machine learning for the application of 6G communication system by achieving 3.186 mean square error and 82% of detection accuracy^30^. The ultra-wideband antenna has been presented for next-generation communication system applications with 11.87 GHz bandwidth^31^. The IoT system operated antenna has been designed with a prediction accuracy of 77.4% in machine learning^32^. The ultra-wideband of 2.9 GHz to 16.37 GHz has been achieved through novel antenna design^33^.

The literature investigated so far shows that most of the designs are applicable for applications related to communications, Wi-fi, etc. There is a strong and urgent need for a high-speed communication MIMO antenna that can operate at high speed. We have proposed a novel antenna design using a slotted O-shape compact design, which not only gives good gain and bandwidth but also shows good diversity performance. The machine learning optimization and parameter optimization are also considered for the improvement of the antenna parameters. The design, results and optimization are discussed in detail in the upcoming sections.

## Design and analysis

**Fig. 1:**
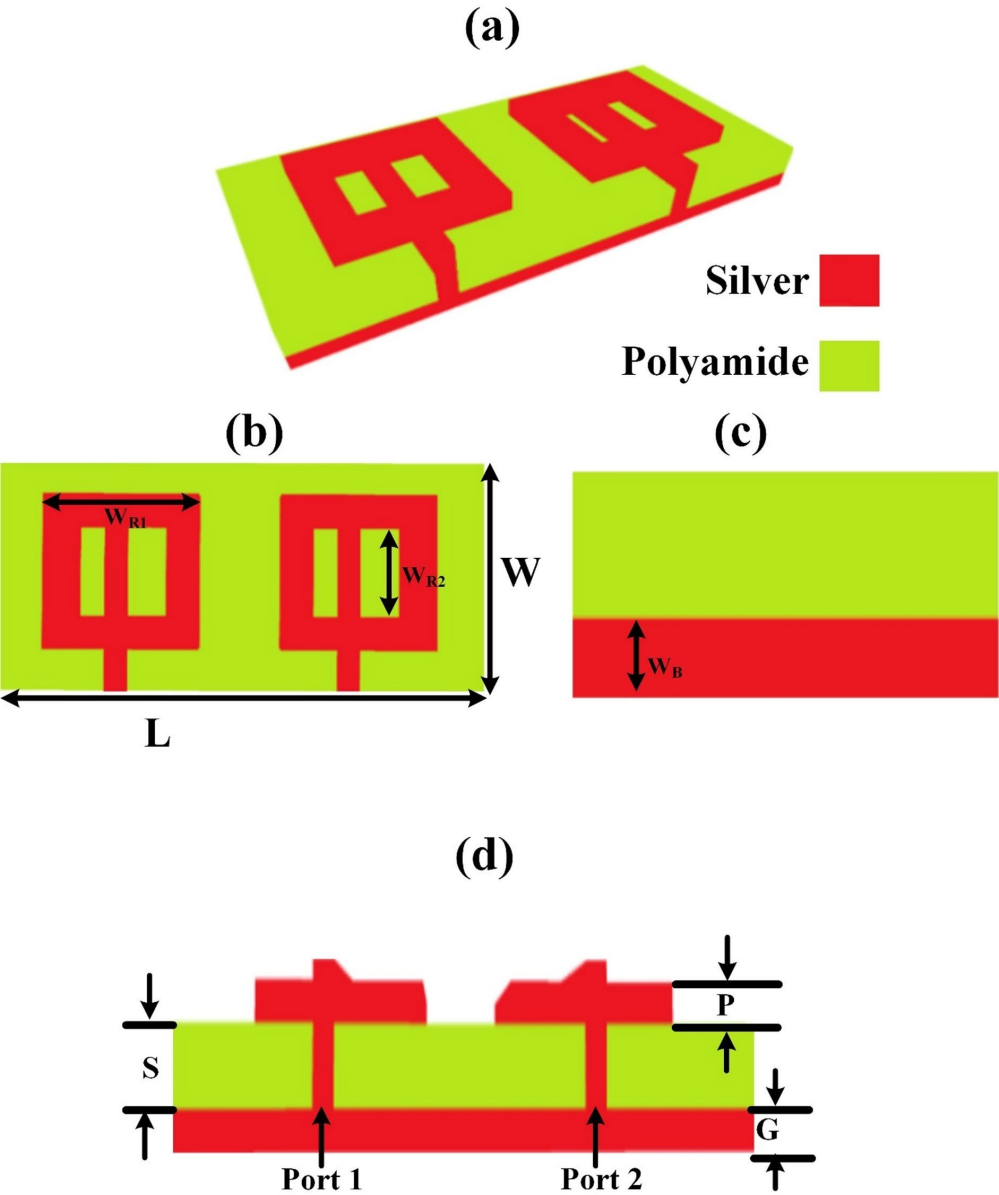
Metamaterial MIMO antenna design with its different views. The Slotted O-shape design is presented. The different structural sizes are *P* = 0.5 μm, G = 1 μm, S = 1.6 μm, L = 110 μm, W = 55 μm, WR1 = 38 μm, WR2 = 25 μm, WB = 25 μm.

The MIMO antenna design is presented in this section in detail. The design analysis is presented in Fig. 1, where the MIMO rectangular patch is presented with a slotted O-shaped design. The different structural views are presented in this figure to explain the depth of the design. Metamaterials are applied to the simple MIMO patch antenna design by incorporating an O-shaped slotted patch in the design. The O-shape patch induces electric and magnetic resonance based on the silver material of patch design and its size, which is 38 × 35 µm^2^. The O-slot is applied to this patch with two different slots, with one slot slightly bigger compared to the other. The metamaterial effect gives the bandgap, resonance and negative index behaviour. The metamaterial-induced MIMO antenna improves the gain and bandwidth, which is, in a way, utilized for high speed communications. The rectangular two slots introduce resonant behaviour and manipulate the electric field distribution, resulting in negative permittivity and permeability. The metamaterial results given in Sect.  3. The substrate made up of polyamide gives flexibility for the mobile and flexible wireless communication electronics. It also gives thermal stability for high-temperature fabrication. The designed antenna with this flexibility can be used for wearable devices. The silver patch and ground plane give excellent conductivity, which is required for the high gain and broad bandwidth. The different geometrical parameters of the design are: *P* = 0.5 μm, G = 1 μm, S = 1.6 μm, L = 110 μm, W = 55 μm, WR1 = 38 μm, WR2 = 25 μm, WB = 25 μm. The ground plane is etched which gives additional electromagnetic interactions like stronger field localization, improved coupling and enhanced resonance behaviour. The ground plane is etched to 25 μm.

**Fig. 2:**
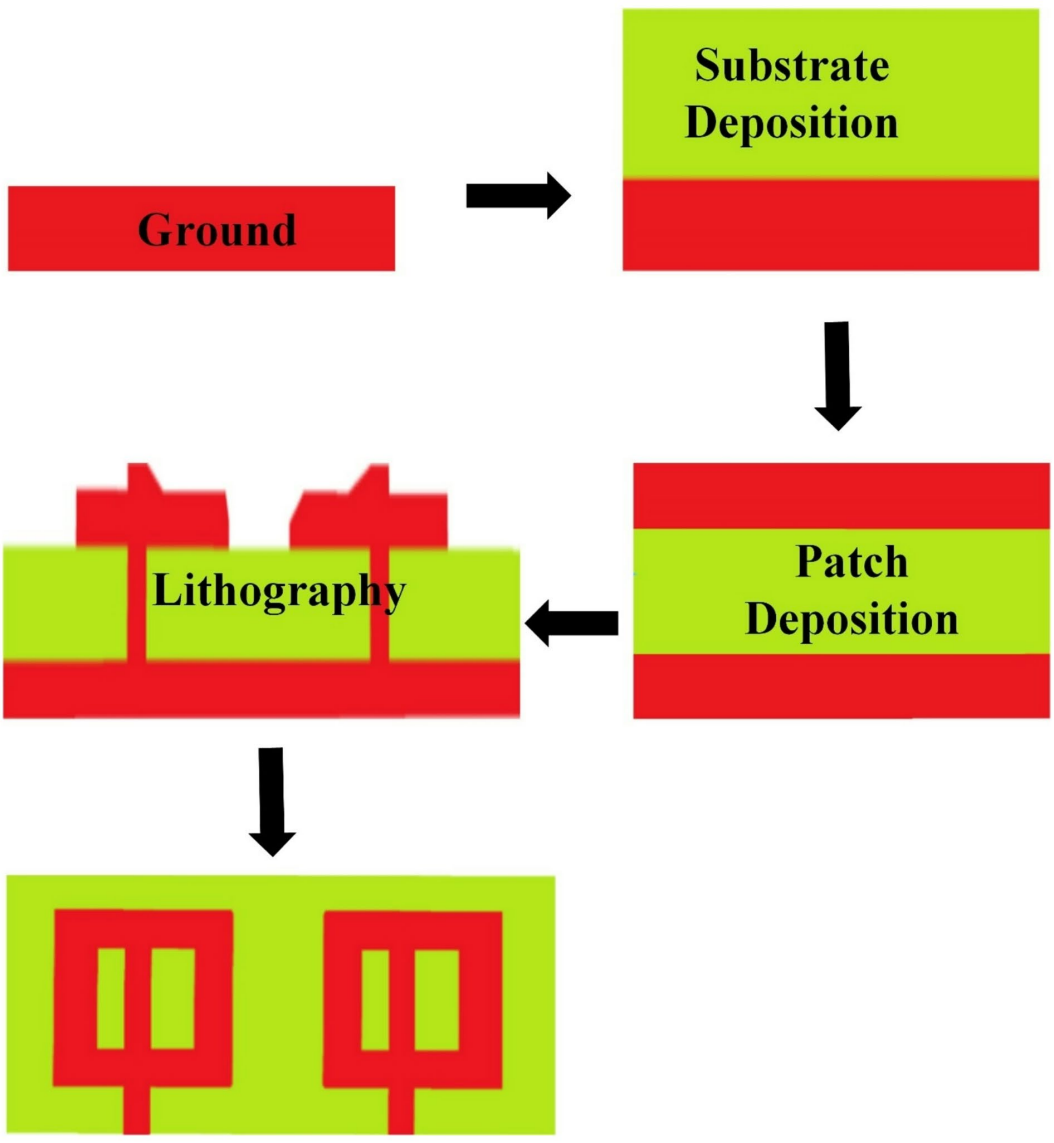
Fabrication approach using deposition and lithography techniques.

The MIM layers are fabricated, and their fabrication approach is presented in Fig. 2. The layer-by-layer fabrication is visible in the figure. The MIM structure is fabricated by sequentially depositing a bottom silver ground layer onto a polyamide substrate using e-beam evaporation. An O-shaped slot is then etched into the ground plane using standard photolithography, followed by wet chemical etching. A thin polyamide insulating layer is applied where necessary and thermally cured. Subsequently, a top silver layer is deposited and patterned to form the O-shaped slotted metamaterial patch using a lift-off process. This layered approach ensures strong capacitive and inductive coupling between the top and bottom metal layers, critical for resonance and negative permittivity behavior. The entire structure is annealed at low temperature to enhance silver conductivity while maintaining substrate flexibility.

.

### Metamaterial analysis

Metamaterial has remarkable properties which can be used for the THz MIMO antenna designs. The metamaterial antenna parameters are described using Eqs. (1–5)^34^. The equations can be implemented by applying the values of S-parameters.

1 $$\:Z=\pm\:\sqrt{\frac{{\left(1+{s}_{11}\right)}^{2}-{s}_{21}^{2}}{{\left(1-{s}_{11}\right)}^{2}-{s}_{21}^{2}}}$$

2 $$\:\:{e}^{in{k}_{0}d}=\frac{{s}_{11}}{1-{s}_{11}\frac{2-1}{2+1}}$$

3 $$\:n=\frac{1}{{k}_{0}d}\left\{\right[ln{e}^{in{k}_{0}d}\left)\right]{\prime\:}{\prime\:}+2m\pi\:\}-i[ln\left({e}^{in{k}_{0}d}\right)\left]{\prime\:}\right]$$

4 $$\:\epsilon\:=\frac{n}{z}$$

5 $$\:\mu\:=nz$$

## Results and discussion

**Fig. 3:**
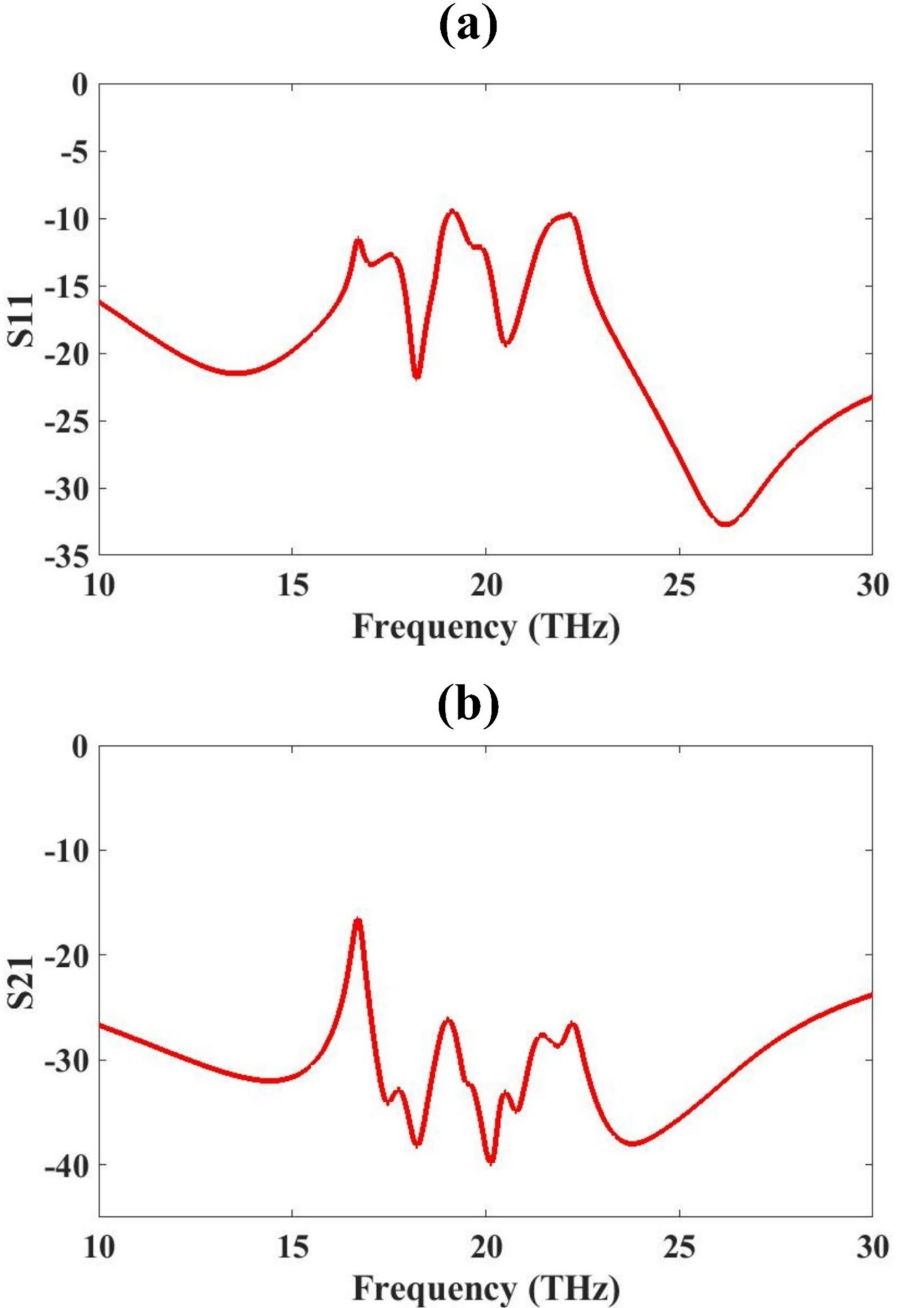
O-shape slotted metamaterial design S-parameter results for 10 to 30 THz range (**a**) S_11_ (**b**) S_21_.

The proposed O-shaped slotted metamaterial-based MIMO antenna was modelled and simulated using COMSOL Multiphysics. The simulated S-parameter results of the proposed O-shaped slotted metamaterial-based MIMO antenna demonstrate promising characteristics for high-speed applications. The S-parameter result is presented in Fig. 3 for both S-parameters. The investigation range is 10 THz to 30 THz. As visible in the figure for the whole range, both S-parameter values are below − 10 dB. The S_11_ parameter exhibits multiple resonances between 17 THz and 25 THz, with reflection coefficients below − 20 dB, indicating efficient multiband operation and strong impedance matching. Meanwhile, the S_21_ response remains lower than − 20 dB for entire frequency range, with values dipping below − 40 dB at key frequencies, suggesting excellent port isolation. These results confirm that the metamaterial design not only enhances bandwidth but also significantly reduces mutual coupling between antenna elements. Such properties are essential for high-capacity, low-interference MIMO systems operating in the terahertz spectrum envisioned for next-generation high-speed networks.

**Fig. 4:**
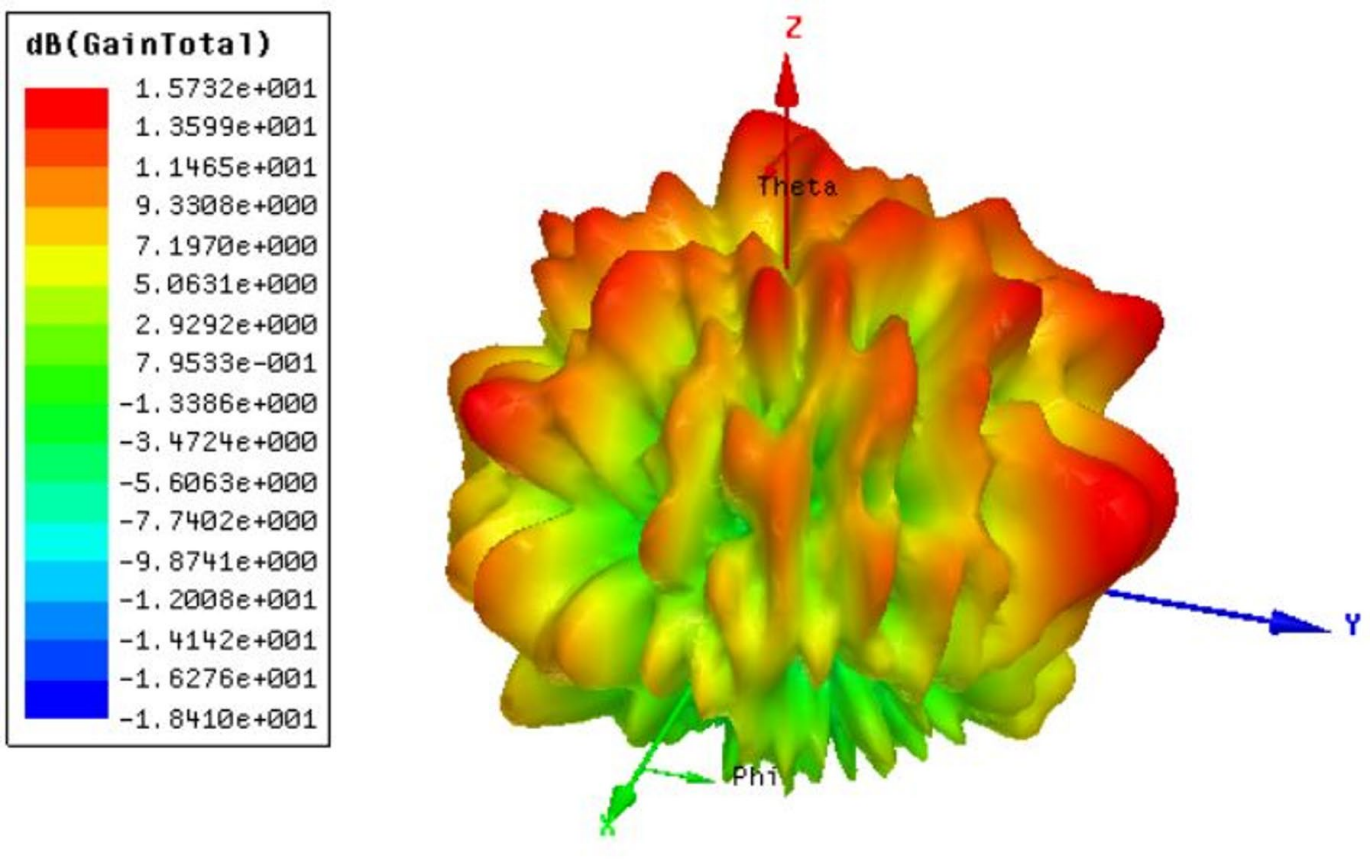
O-shape slotted metamaterial design gain polar plot response. The highest gain is 15.7 dBi.

Figure 4 presents the 3D gain polar plot of the proposed O-shaped slotted metamaterial-based MIMO antenna, simulated using COMSOL Multiphysics. The radiation pattern exhibits a multi-lobed structure, with the maximum gain reaching approximately 15.73 dB. The presence of several prominent lobes in various directions highlights the antenna’s excellent spatial diversity, which is critical for MIMO performance in high speed communication systems. This quasi-omnidirectional radiation pattern enables robust signal transmission and reception in multipath environments. The complex distribution is attributed to the metamaterial-enhanced current paths and mutual coupling effects between the MIMO elements. The wide angular coverage and high gain validate the antenna’s suitability for high-frequency (THz) applications, where wideband, multi-user, and high-throughput capabilities are essential.

### Parametric analysis

**Fig. 5:**
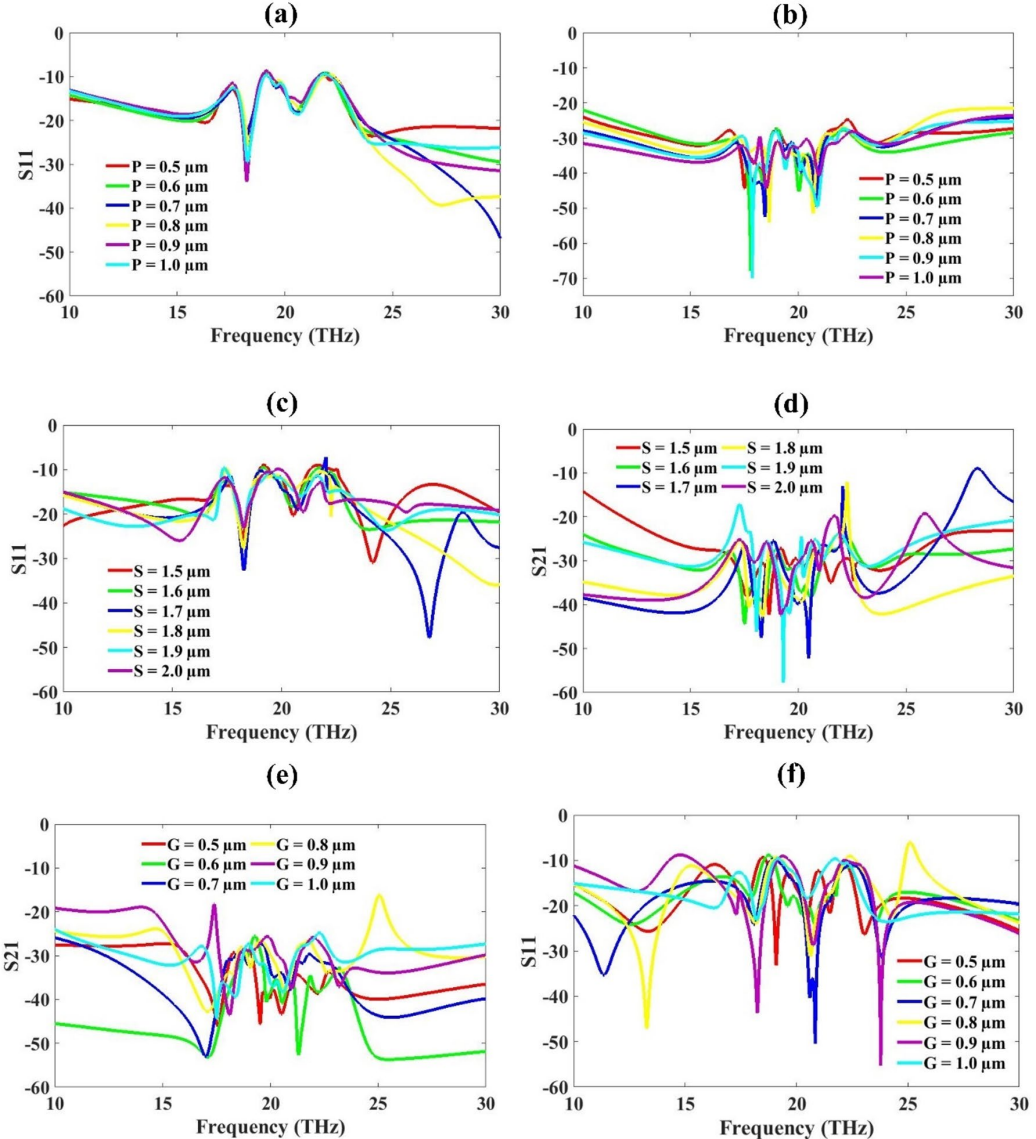
O-shape slotted metamaterial design S-parameter response, Patch variation (**a**) S11 (**b**) S21, Substrate variation (**c**) S11 (**d**) S21, Ground Plane variation (**e**) S11 (**f**) S21.

**Fig. 6:**
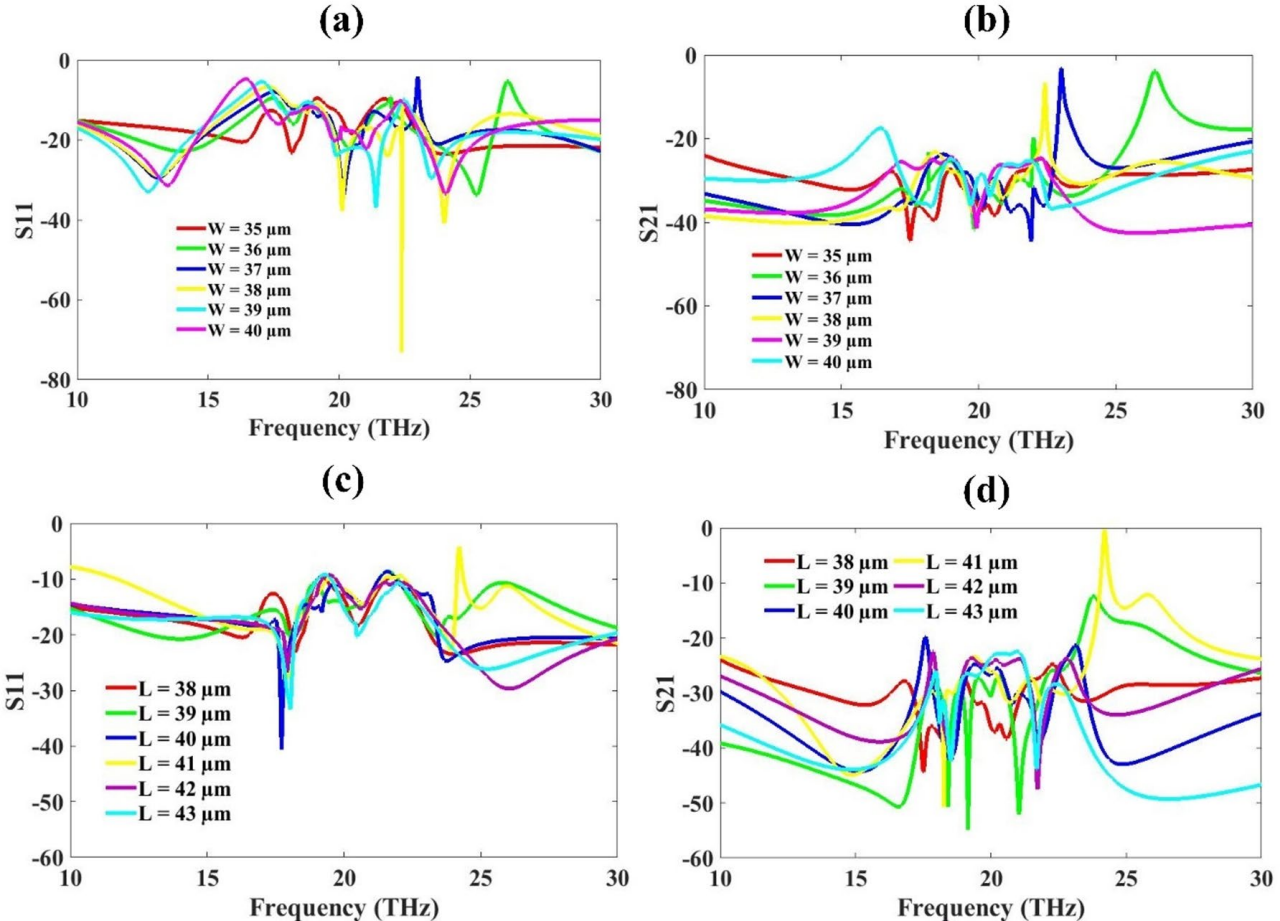
O-shape slotted metamaterial design S-parameter response, Width variation (**a**) S11 (**b**) S21, Length variation (**c**) S11 (**d**) S21.

Parametric optimization was carried out to evaluate the results of the proposed MIMO metamaterial antenna design to variations in structural parameters. Key variable including the substrate and patch thicknesses, ground plane thickness, substrate length and width. Ground plane width. All these variables are systematically varied while monitoring the S-parameter results in Figs. 5 and 6.

The results for thickness variation are given in Fig. 5. The patch thickness P is varied from 0.5 to 1 μm. In S_11_, Lower values of P (0.5–0.7 μm) exhibit deeper notches, indicating better impedance matching (S_11_<-10 dB) across multiple resonances, particularly around 15–20 THz. In S_21_, *P* = 0.5 μm and 0.6 μm show stronger transmission dips, which may indicate effective resonance coupling or absorption at those frequencies. This suggests that the optimized values can be 0.5–0.6 μm and to make the design compact, we have selected the optimized value to be 0.5 μm for patch design. This value balances compactness with enhanced reflection and transmission performance at target resonance bands.

The substrate thickness S varied from 1.5 to 2 μm. In S_11_ response, At S = 1.6–1.7 μm, multiple deep notches appear around 15–25 THz, indicating good resonance formation. In S_21_ response, S = 1.6–1.8 μm demonstrates sharper dips, showing strong filtering or absorption features. So optimized values are 1.6 μm. These values offer a good trade-off between dielectric loading and bandwidth, improving device resonance sharpness and performance. Ground plane thickness is varied from 0.5 to 1 μm. The S_11_ and S_21_ results show that the optimized thickness is 1 μm.

Figure 6 shows the parametric analysis of a metamaterial-based MIMO antenna structure, focusing on the variations in width (W) and length (L) of the metamaterial resonator. The plots evaluate the antenna’s performance using S-parameters (S_11_ and S_21_) over a frequency range of 10–30 THz. The length is varied from 38 to 43 μm and the width is varied from 35 to 40 μm. The response was presented. The length and width value results do not have much difference as per the requirement of high speed communication applications. It is better to select a shorter length and width, which will lower the size and cost of the design.

### Diversity parameter analysis

**Fig. 7:**
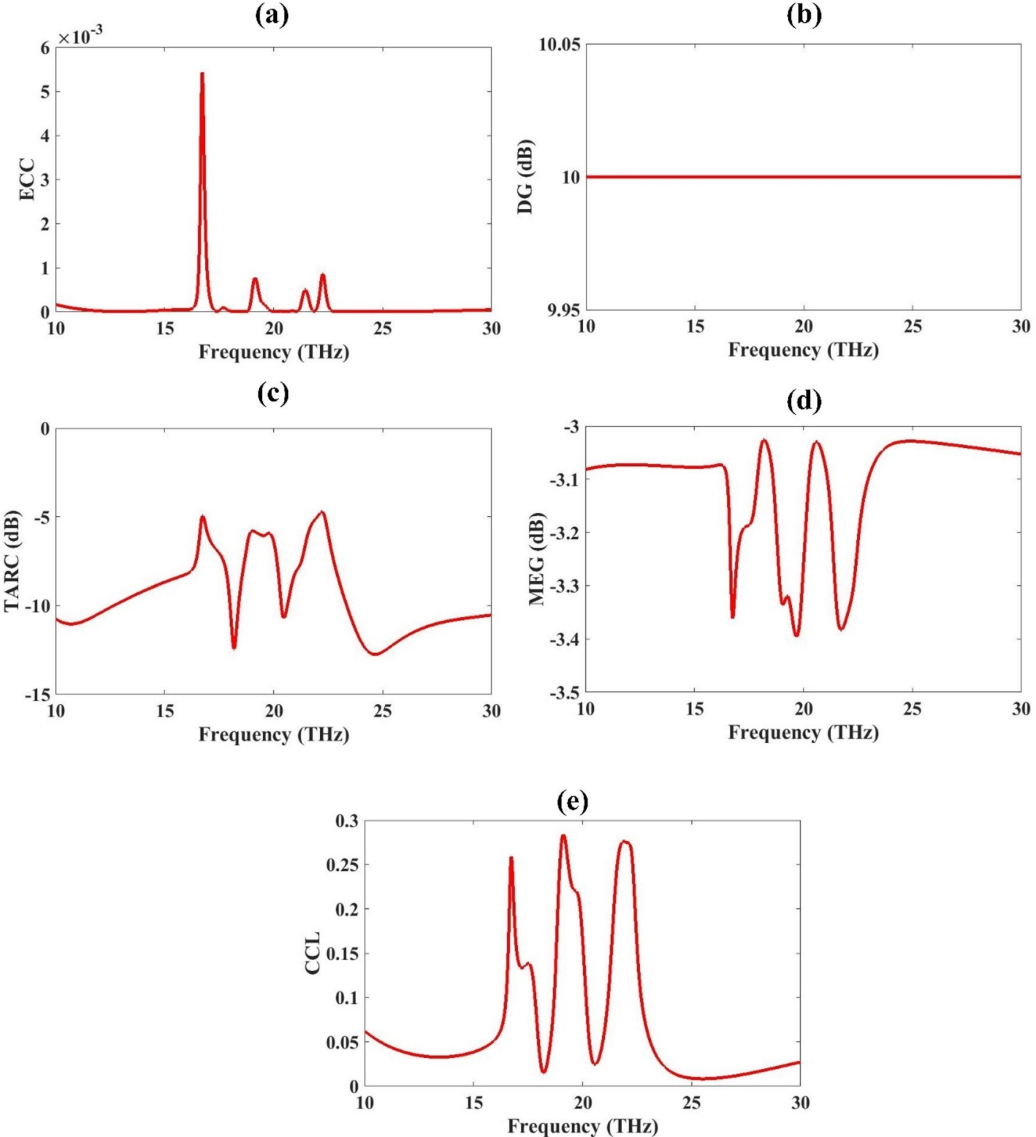
MIMO parameter analysis. (**a**) ECC is near zero (**b**), DG is 10 dB (**c**) TARC is less than − 5 dB for the whole range (**d**), MEG is lower than 3dB and (**e**) CCL is lower than 0.3 bits/Hz.

Figure 7 presents critical MIMO performance parameters for a THz metamaterial-based antenna, evaluated over the 10–30 THz frequency range. These metrics are essential for assessing diversity, isolation, efficiency, and correlation in MIMO design. The ECC is near to zero for 10 to 30 THz range. This shows the better MIMO antenna performance^35^. DG depends mainly on the value of ECC. Lower ECC values near zero give the DG values near 10dB which is required for better MIMO antenna performance^36^. DG is perfectly flat at 10 dB, confirming the antenna’s ideal diversity gain and consistent performance across the entire THz range. TARC measures the combined reflection of all antenna ports when multiple ports are simultaneously excited with various signal phases^37^. TARC dips below **−** 10 dB in key frequency bands (~ 14–26 THz), suggesting excellent impedance matching and low active reflection, which is critical in practical MIMO operation. and low active reflection, which is critical in practical MIMO operation. MEG quantifies the average received power of an antenna in a multipath environment, considering the radiation pattern, polarization, and propagation conditions^38^. MEG remains between **−** 3.0 and **−** 3.4 dB, showing balanced performance and minimal gain mismatch between MIMO elements. CCL is a critical parameter used to evaluate the diversity performance and isolation between antenna elements in a multi-antenna system^39^. CCL is consistently below 0.3, ensuring low channel degradation and high data throughput, making the antenna system efficient for high-speed THz MIMO communication. This analysis confirms the proposed antenna as a robust MIMO candidate for high-speed THz systems, offering low correlation, high diversity, and efficient transmission performance.

### Metamaterial parameters

**Fig. 8:**
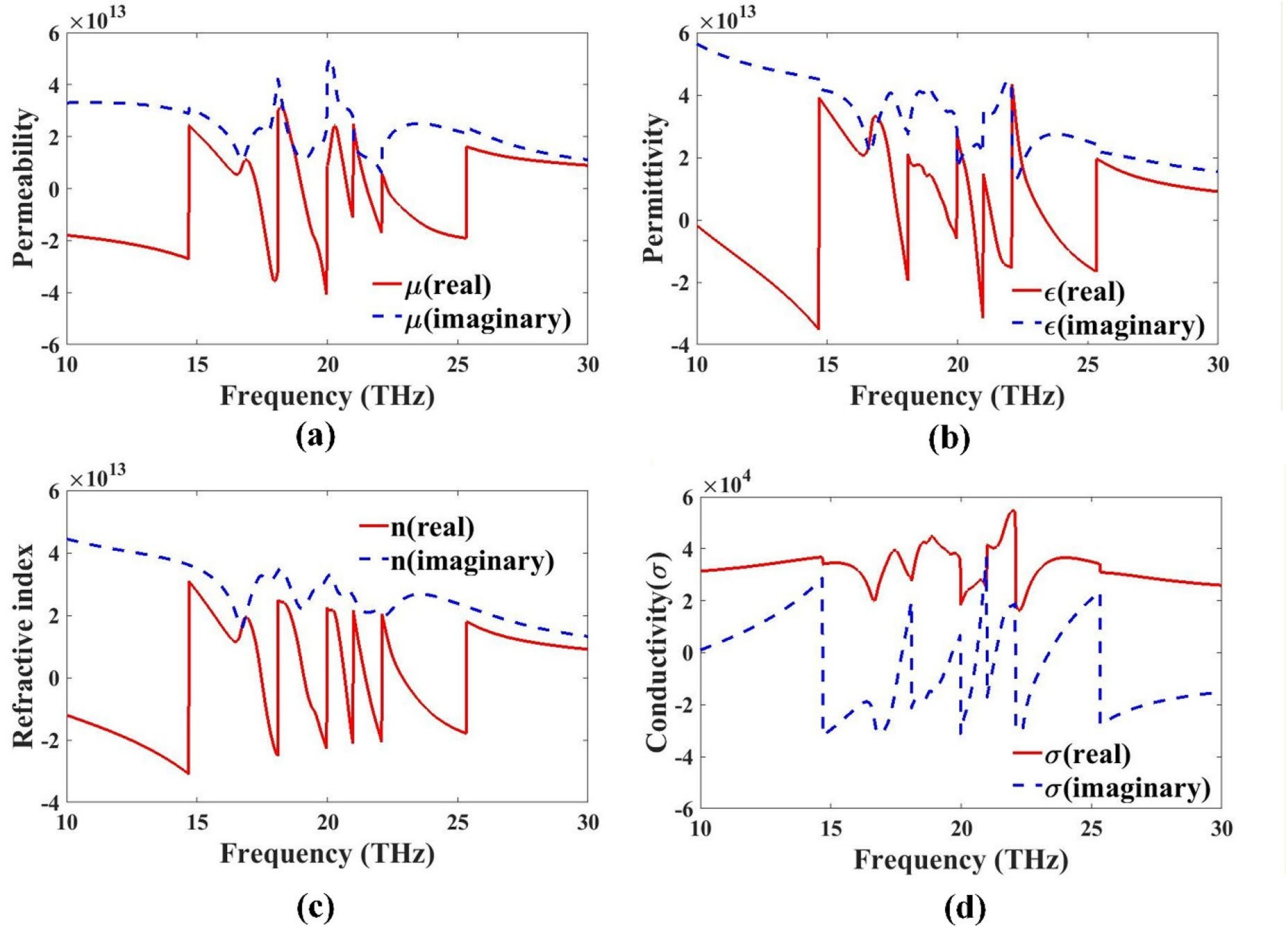
Metamaterial parameter analysis. (**a**) Permeability-µ, (**b**) permittivity-ε, (**c**) refractive index- n, and (**d**) conductivity-σ.

Figure 8 presents the metamaterial design for evaluating its electromagnetic behavior. These parameters are extracted from the S-parameters using retrieval methods^40^. The real part is negative at several bands, especially near 15–22 THz, suggesting epsilon-negative (ENG) properties. The imaginary part again shows material losses due to dielectric behavior. The metamaterial exhibits epsilon-negative behavior, contributing to double-negative (DNG) regions when both ε and µ are negative. The real part of n dips into the negative region at multiple frequencies (especially between 15 and 22 THz). This confirms the left-handed (negative index) behavior of the metamaterial. The imaginary part denotes absorption; higher peaks suggest resonance-related losses. The structure supports a negative refractive index in several bands, confirming metamaterial nature. The real part shows how well the structure conducts current. Variations and peaks near resonances (~ 18–23 THz) indicate frequency-dependent conduction, typical of metamaterials. The imaginary part represents reactive losses and energy storage. The structure exhibits dynamic conductivity, influenced by resonances, useful for THz filter, absorber, or sensor applications.

## Machine learning optimization

Machine learning for the developed work on the current antenna, using the applied method of linear regression, has been optimized as follows. In the ML section, each layer height analysis allows us to study the output efficiencies with both actual and predicted values for S11 sections. The current ML results show R² values with a minimum mean square error rate, and the test size is 0.25.

**Fig. 9:**
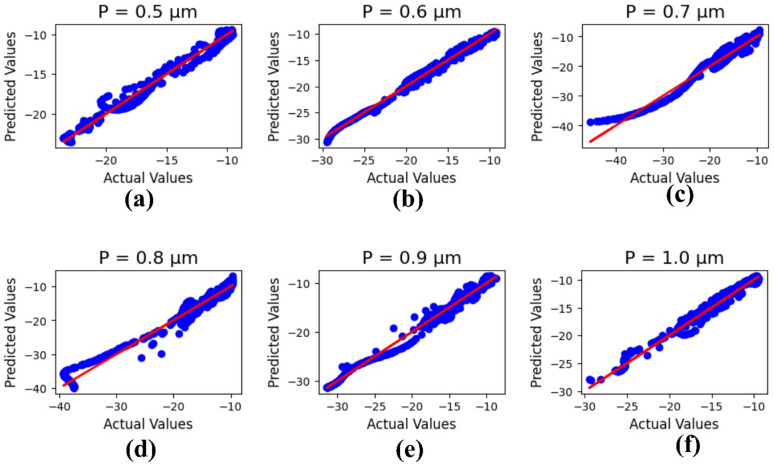
Machine learning optimization for the varying patch height with parametric values (µm) of (**a**) 0.5, (**b**) 0.6, (**c**) 0.7, (**d**) 0.8, (**e**) 0.9, and (**f**) 1.0.

Figure 9 presents the machine learning results for the patch height ‘P,’ with parameter values (µm) varying from 0.5 to 1.0. The outputs refer to the actual and predicted values, and the R² results are 0.97, 0.99, 0.96, 0.96, 0.98, and 0.98, with a mean square error of 0.27175.

**Fig. 10:**
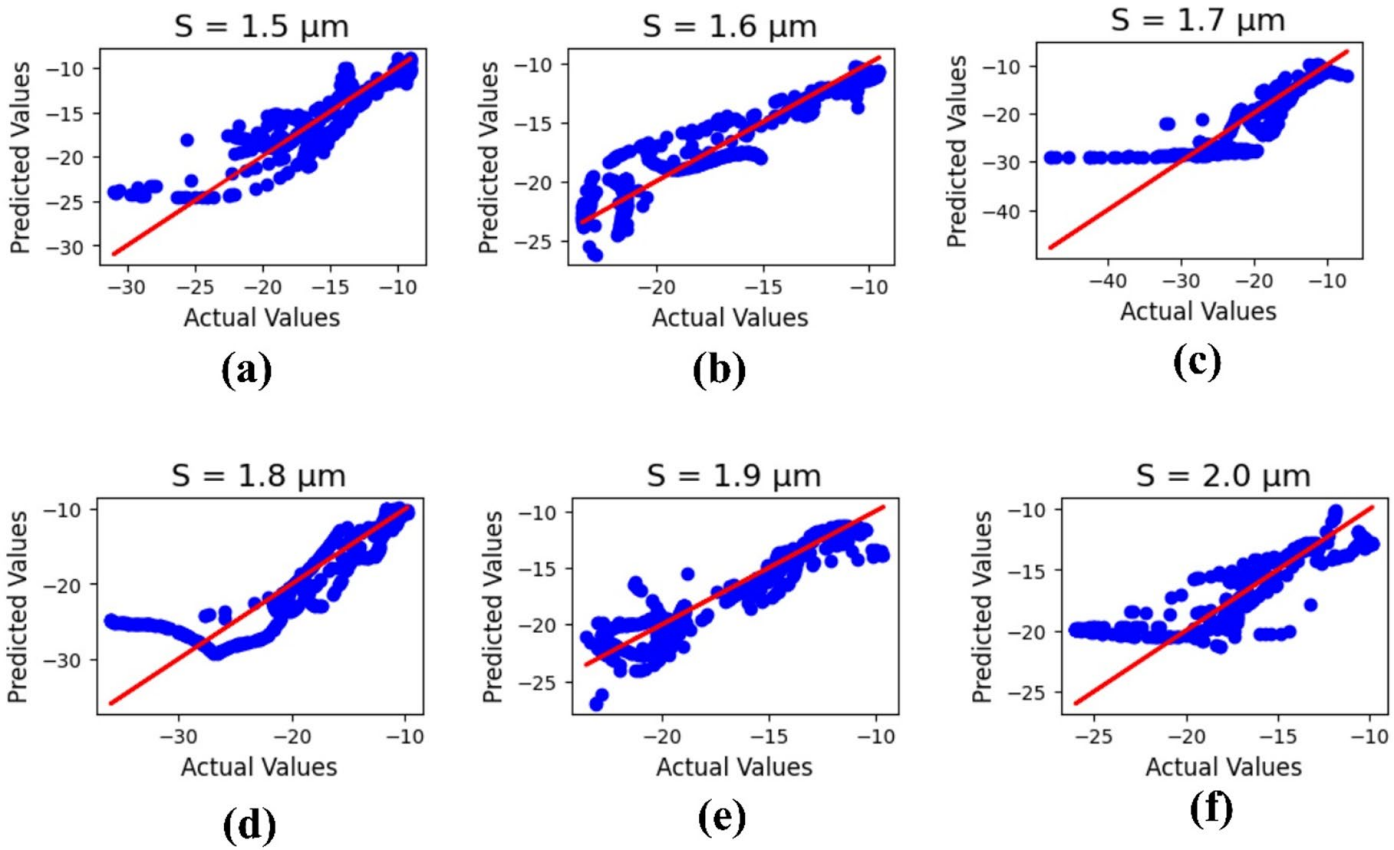
Machine learning optimization for the varying substrate height with parametric values (µm) of (**a**) 1.5, (**b**) 1.6, (**c**) 1.7, (**d**) 1.8, (**e**) 1.9, and (**f**) 2.0.

Figure 10 configures the machine learning results for the substrate height ‘S,’ with parameter values (µm) varying from 1.5 to 2.0. The outputs refer to the actual and predicted values, and the R² results are 0.76, 0.85, 0.73, 0.72, 0.82, and 0.65, with a mean square error of 2.43605.

**Fig. 11:**
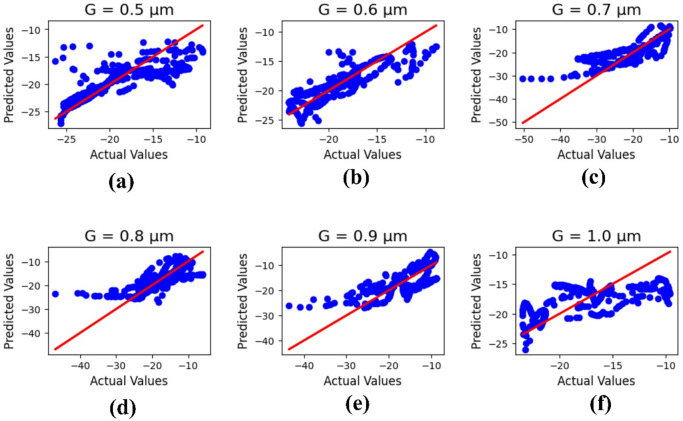
Machine learning optimization for the varying ground height with parametric values (µm) of (**a**) 0.5, (**b**) 0.6, (**c**) 0.7, (**d**) 0.8, (**e**) 0.9, and (**f**) 1.0.

Figure 11 shows the machine learning results for the ground height ‘G,’ with parameter values (µm) varying from 0.5 to 1.0. The outputs refer to the actual and predicted values, and the R² results are 0.59, 0.75, 0.56, 0.57, 0.52, and 0.44, with a mean square error of 3.16409.

**Fig. 12:**
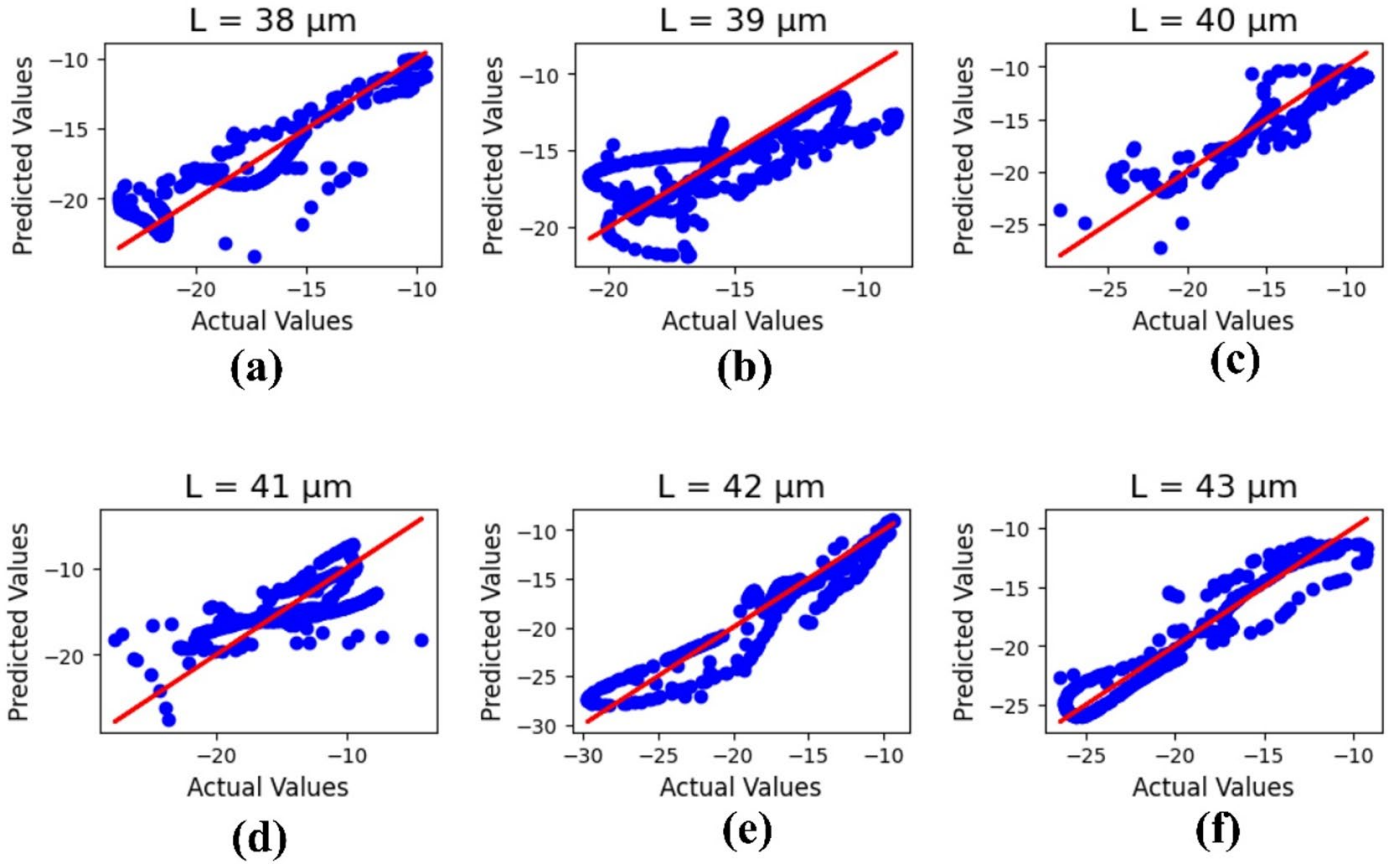
Machine learning optimization for the varying antenna length with parametric values (µm) of (**a**) 38, (**b**) 39, (**c**) 40, (**d**) 41, (**e**) 42, and (**f**) 43.

Figure 12 illustrates the machine learning results for the antenna length ‘L,’ with parameter values (µm) varying from 38 to 43. The outputs refer to the actual and predicted values, and the R² results are 0.82, 0.52, 0.85, 0.49, 0.88, and 0.89, with a mean square error of 3.93133.

**Fig. 13:**
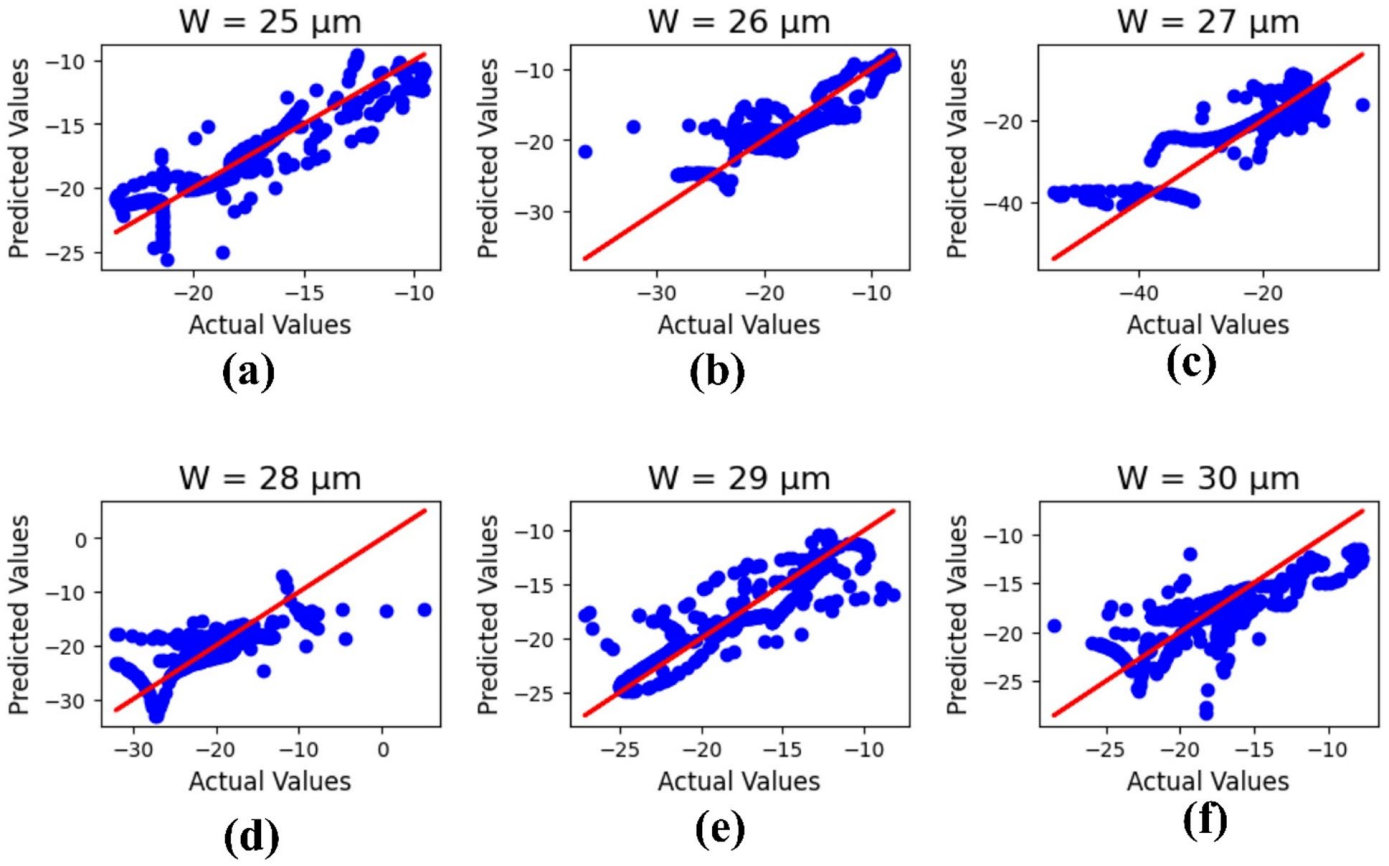
Machine learning optimization for the varying antenna width with parametric values (µm) of (**a**) 25, (**b**) 26, (**c**) 27, (**d**) 28, (**e**) 29, and (**f**) 30.

Figure 13 illustrates the machine learning results for the antenna width ‘W,’ with parameter values (µm) varying from 25 to 30. The outputs refer to the actual and predicted values, and the R² results are 0.84, 0.71, 0.76, 0.74, 0.72, and 0.59, with a mean square error of 2.23223.

The comparison analysis of the results of structure response with other responsees is shown in Table 1. This analysis identifies how our design gives better gain and bandwidth with a smaller size compared to other designs, making our design overall effective for high-speed THz applications.

**Table 1:** Comparison of different antenna parameters of our design with other references.

References	Size (µm^2^)	Bandwidth (THz)	Gain (dB)	Application
^41^	110 × 130	0.78	7.5	Wireless communication systems
^42^	1000 × 1400	9.6	19	Terahertz application
^43^	2000 × 1000	76	10.43	Terahertz application
^44^	1000 × 1400	9.67	19	Terahertz application
^13^	–	0.13	8	IoT networks
^45^	13 × 26	18.18	1,5	Mutual coupling reduction
^46^	125 × 125	9.3	-	Terahertz application
^47^	800 × 600	5.71	7.934	Photonic crystal in THz
^48^	130 × 85	0.6	7.23	6G/IoT application
^49^	822 × 280	0.116	13.6	Wireless communication systems
^50^	600 × 300	0.4	5.49	Terahertz application
^51^	800 × 600	9	9.5	Terahertz application
^52^	500 × 500	–	8.36	Wireless communication systems
^53^	360 × 220	0.6	11.8	THz communication
^54^	103 × 80	1.77	14.44	6G communication
^55^	800 × 1170	14.8	-	THz communication
^56^	90 × 30	6	12.38	6G communication
^57^	122 × 51	8.3	10.34	THz communication
Two-port MIMO antenna design	110 × 55	20	15.7	Terahertz application

## Conclusion

The Metamaterial antenna form with an O-shaped design, demonstrates significant enhancements in performance metrics essential for next-generation wireless systems, including TWPAN. The parametric optimization of structural elements such as patch height, substrate thickness, and ground plane configuration has led to improved impedance matching, isolation, gain, and bandwidth. The antenna exhibits low mutual coupling, and good gain for desired frequency range. Additionally, the metamaterial inclusion supports miniaturization without compromising radiation efficiency. The design also achieves high DG and low ECC. The CCL is also less than 0.3 bits/Hz, affirming its suitability for MIMO applications. The gain of the design is improved to 15.7 dBi with an overall bandwidth of 20 THz. The machine learning algorithm gives the higher behaviour prediction with R^2^ value of 0.99. Overall, the optimized antenna structure meets the stringent requirements of high data throughput, small size, ultra-broad bandwidth which can be used for future TWPAN systems. The presented work with advanced material analysis, as well as a machine learning approach, helps for further fabrication in various applications.

## Data Availability

The data supporting the findings in this work are available from the corresponding author with reasonable request.
